# Interleukin-1β sequesters hypoxia inducible factor 2α to the primary cilium

**DOI:** 10.1186/2046-2530-2-17

**Published:** 2013-12-13

**Authors:** Angus KT Wann, Clare L Thompson, J Paul Chapple, Martin M Knight

**Affiliations:** 1 Institute of Bioengineering and School of Engineering and Materials Science, Queen Mary University of London, Mile End, London, E1 4NS, UK; 2 Centre for Endocrinology, William Harvey Research Institute, Barts and the London School of Medicine and Dentistry, Queen Mary University of London, London, UK

**Keywords:** Primary cilium, Hypoxia-inducible factor, Cytokine, Interleukin-1, Inflammation, Chondrocyte, Prolyl hydroxylase, Intraflagellar transport

## Abstract

**Background:**

The primary cilium coordinates signalling in development, health and disease. Previously we have shown that the cilium is essential for the anabolic response to loading and the inflammatory response to interleukin-1β (IL-1β). We have also shown the primary cilium elongates in response to IL-1β exposure. Both anabolic phenotype and inflammatory pathology are proposed to be dependent on hypoxia-inducible factor 2 alpha (HIF-2α). The present study tests the hypothesis that an association exists between the primary cilium and HIFs in inflammatory signalling.

**Results:**

Here we show, in articular chondrocytes, that IL-1β-induces primary cilia elongation with alterations to cilia trafficking of arl13b. This elongation is associated with a transient increase in HIF-2α expression and accumulation in the primary cilium. Prolyl hydroxylase inhibition results in primary cilia elongation also associated with accumulation of HIF-2α in the ciliary base and axoneme. This recruitment and the associated cilia elongation is not inhibited by blockade of HIFα transcription activity or rescue of basal HIF-2α expression. Hypomorphic mutation to intraflagellar transport protein IFT88 results in limited ciliogenesis. This is associated with increased HIF-2α expression and inhibited response to prolyl hydroxylase inhibition.

**Conclusions:**

These findings suggest that ciliary sequestration of HIF-2α provides negative regulation of HIF-2α expression and potentially activity. This study indicates, for the first time, that the primary cilium regulates HIF signalling during inflammation.

## Background

The solitary primary cilium is a tubulin-based organelle constructed by the majority of cell types upon exit from the cell cycle. The cilium has emerged as fundamental to, or a subtle tuner of, cellular signalling such as the hedgehog [1-3], wnt [4,5], platelet-derived growth factor (PDGF) [6], insulin growth factor (IGF) [7] and transforming growth factor (TGF) pathways [8]. As such, it is implicated in many facets of cell biology, exerting influence over the cell cycle [9], differentiation [8,10,11] and mechanobiology [12-15]. The cilium is consequently critical to the development and health of many tissue types. The cilium’s tubulin structure and contents are maintained and supplied by intraflagellar transport (IFT) proteins, which shuttle proteins into the axoneme towards the tip and back to the basal body at the cilia base [16,17]. Cilia structure, notably length, and function are inter-related, as both are largely defined by ciliary trafficking. This relationship is highlighted by small molecule approaches and genetic mutations in IFT and associated proteins which regulate cilia trafficking producing a change in cilia length and function [18-23]. Thus cilia length, which is altered in many physiological and pathological contexts, provides an indicator of ciliary trafficking.

Inflammation is often characterised by the elevation of cytokines. The quintessential pro-inflammatory cytokine Interleukin-1 (IL-1) canonically triggers a broad spectrum of physiological consequences. These inflammatory signals serve resolution and repair but also represent a component at the heart of many diseases, from cancers to arthritis. IL-1 has been shown to influence one or both α-subunits of the hypoxia inducible factors (HIFs) [24-26], however differences in the responses occur and are most likely due to different cell types or experiment conditions. The HIFs are transcription factors with a very broad biological significance to many cell and tissue types [27]. Canonical regulation of HIF abundance is governed after transcription and translation in part due to the action of oxygen sensitive enzymes, the hif-α prolyl hydroxylases. These enzymes tag HIFs prior to Von Hippel Lindau protein (vHL) ubiquitination and destruction in the proteosome. Hypoxia maintains HIFα protein expression through inhibition of prolyl hydroxylases and IL-1 is suggested to effect subunit expression at the level of transcription and in a similarly post-translational fashion [26]. Relatively little is known about regulatory mechanisms in HIF signalling, especially with regards to HIF-2 but other putative mechanisms for the maintenance of HIF expression include stabilisation through binding of the molecular chaperone heat shock protein, HSP90 [28]. Recent studies have indicated that IL-1β increases HIF-2α expression in murine and rabbit chondrocytes and by doing so activates, among other targets, nitric oxide synthase 2 and prostaglandin endoperoxide synthase-2 [29]. Somewhat in disagreement with this, studies using human chondrocytes have carefully documented the roles of HIF proteins, in anabolic (increases in aggrecan expression) and anti-catabolic responses [30]. In other contexts such as cancer, HIF-2α has been shown to directly activate prostaglandin E_2_ (PGE_2_) signalling [31].

Previous work in our group has shown primary cilia are required for both mechanically-induced upregulation of aggrecan synthesis [15] and IL-1-induced PGE_2_ and nitric oxide (NO) release [32]. We also observed cilia elongation in response to IL-1. Interestingly, alteration in HIF expression by hypoxia or pharmacological mimics has also been shown to influence primary cilia length [33,34] and activate the hedgehog pathway [35]. The rationale for the current studies was therefore to examine the interaction between IL-1 and HIF and elucidate the role of the primary cilium and cilia elongation in this interaction.

Given the established roles for both HIFs [36] and primary cilia [15,32,37-40] in cartilage physiology and inflammatory arthritis [29], chondrocytes represent an apt cell model with physiological and pathological relevance. Furthermore the quiescent nature of chondrocytes makes them ideal for studying primary cilia structure-function since cilia are only expressed outside of the cell cycle.

We show here that IL-1 exposure results in dynamic alteration in cilia length indicative of altered trafficking. This is associated with both a transient increase in HIF-2α expression and also, intriguingly, with cilia localised accumulation of HIF-2α. We demonstrate that prolyl hydroxylase inhibition also results in ciliary elongation and a more pronounced recruitment of HIF-2α to the ciliary base and sequestration to the ciliary axonome. IL-1-induced cilia elongation and HIF2α ciliary localisation is not mediated by the transcriptional activity of HIFα or the increase in HIF-2 α expression. Instead we propose that elongation drives ciliary sequestration leading to negative regulation of HIF-2α expression and activity. These data reveal a completely new relationship between HIFs and the primary cilium in inflammation, which may have important implications for diseases such as arthritis and cancer.

## Methods

### Pharmacological and biological reagents and primary antibodies

All reagents were from Sigma Aldrich UK unless stated. Cobalt chloride (CoCl_2_), Trichostatin A (TSA), Y27632 dihydrochloride monohydrate (Y27632); 17-(allylamino)17-demethoxygeldamycin (GA), Dimethyloxallyl glycine (DMOG): Cambridge Bioscience. Human recombinant IL-1β, and Oncostatin-M (ONC-M): both Peprotech, Echinomycin (Ech): Merck Chemicals. The primary cilium axoneme was labelled using mouse anti-acetylated α tubulin (Clone 6-11B-1, Sigma-Aldrich, 1:2,000) and rabbit anti-arl13b (Source bioscience UK, 17711-1-AP, 1:1,000). HIF-1α and HIF-2α were labelled for immunofluorescence and western blot purposes using rabbit anti-HIF-1α (Santa-Cruz, SC-10790, 1:500/1:200) and rabbit anti-HIF-2α (AbCam, ab20654, 1:700). Mouse Anti-β-tubulin (Sigma, T4026, 1:5,000) was used for relative expression.

### Cell sourcing and culture

Bovine and human primary articular chondrocytes were isolated as per previous studies [32]. Cartilage was removed from the metacarpal phalangeal joints of recently slaughtered steers. Human cartilage was obtained from patients undergoing total knee arthroplasty at the Royal London Hospital, Barts and the London NHS Trust, London, UK. This procedure was conducted with ethical approval (East London and The City Research Ethics Committee) and informed patient consent. Cartilage was removed from the femoral condyles and tibial plateaus. The morphology of the cartilage specimens was graded for gross degenerative changes according to the international cartilage repair society classification, and tissue that represented normal (grade 0 or 1) was used for experiments. Cells were isolated by sequential enzymatic digest before culture, for approximately 5 days, at high density (from 80,000 cells.mL^-1^) to form stable, confluent, quiescent (as previously indicated by ki-67 staining) cultures prior to treatments. Primary bovine and human chondrocytes were cultured in low glucose media with 10% serum as described previously, creating the stable conditions best for cilia length studies [32]. The chondrocyte cell line harbouring the hypomorphic mutation in IFT88, as first described in the Oak Ridge Polycystic Kidney (ORPK) mouse model [41,42], were maintained as conditionally immortalised cells (under permissive conditions of 33°C culture and the presence of 10 ng.mL interferon-γ). For all experiments conditional immortalisation was switched off by 3 days non-permissive culture at 37°C without interferon γ and as such used ‘primary’ cells designated wild-type (WT) and ORPK as described both in results here and previously [32]. Quiescent culture, as for bovine primary cells (above), is established before experiments were conducted.

### Pharmacological treatments

Small molecules were added to confluent, monolayer cultures with controls using suitable vehicles. Interleukin-1β was used as previously described [32] at 10 ng.mL^-1^ unless otherwise stated. All other doses are stated throughout.

### Hypoxia study

Confluent cells were cultured for 24 h at 2% oxygen using an oxygen controlled incubator (Binder, Germany). Control cells were maintained at ambient oxygen.

### Immunocytochemistry

Monolayer cultures were fixed with 4% paraformaldehyde at 37°C for 8 min, permeabilised (5 min, 0.5% triton) and blocked (30 min, 5% goat serum). Primary antibodies were incubated in tandem in 0.1% bovine serum albumin-phosphate buffered saline (BSA-PBS) at 4°C overnight or at room temperature for 4 h. After washing, anti-mouse and anti-rabbit alexa-fluor 488 and 594 secondaries (Invitrogen, 1:500) were used in tandem in 0.1% BSA-PBS at room temperature for 1 h. Nuclei were counter-stained with 4',6-diamidino-2-phenylindole (DAPI) (Sigma, 1 μg.mL^-1^) and samples mounted prior to microscopy. Secondary antibody only controls were conducted throughout.

### Western blot analysis

Cell lysates were collected quickly on ice as follows. Preparations were washed once in ice cold PBS containing 50 μM sodium orthovanadate before addition of a lysis buffer of PBS, Roche cocktail inhibitors, 50 μM sodium orthovanadate and 0.1% Igebal (both Sigma). Samples were left on ice for 15 min before scraping and 5 x homogenisation through a 21G needle. Samples were then spun at 13,000 RPM for 15 min at 4°C before supernatant was frozen in liquid nitrogen. For westerns, samples were diluted 1:1 with lamelli buffer and boiled at 100°C for 5 min. Samples of approximately 30 μL, or 50 μg protein as assessed by Bradford assay, were run on a 10% tris (hydroxymethyl)aminomethane-hydrochloride gel before transfer to nitrocellulose membrane. Transfers and loading were checked using ponceau staining. A 1h 5% milk blocking step preceded primary antibody incubations overnight at 4°C. Licor infrared secondarys were incubated at 1:15,000 for 1 h at room temperature preceded and followed by 3 × 10 min washes in 0.1% PBS Tween. Relative protein expression was established by quantitative analysis of specific bands (Licor Odyssey integrated intensity values) and expressed relative to β-tubulin. Linearity was tested by standard curve using serial dilutions of samples probed for β-tubulin.

### PGE_2_ ELISA

Quantitative immunoassay (R&D Systems, UK) was used to quantify media PGE_2_ concentrations in media immediately following 24 h DMOG treatment as previously described [32]. Absorbance was measured at 450 nm. Results were corrected for non-specific binding and read from a PGE_2_ standard curve fitted in GraphPad prism 5.

### Imaging

Cilia imaging was conducted based on protocols described in full elsewhere [32]. To review briefly, an oil immersion x63 objective and scanning confocal microscopy (SP2 Leica) were used to produce confocal serial sections for z stack reconstructions of monolayer fields (pixel size = 0.23 μm or smaller for single cilia images). From reconstructed z projections, cilia lengths were measured in Image J. Secondary only controls were conducted to ensure thresholds for co-localisation studies.

### Statistics

Data manipulations and analysis were conducted using GraphPad Prism 5. For cilia length measurements Mann–Whitney U tests were performed due to the naturally skewed nature of the data. Cilia length data are presented in box and whisker format where the centre line is the median, the box marks 25th-75th percentiles and whiskers are 10th-90th percentiles. For all cilia length data *n* is >100 cilia for each group. Experiments were repeated at least twice, with three coverslip replicates and cilia length data pooled. Cells were isolated from at least six animals. For quantitative western blots and qPCR unpaired t-tests were employed and means with S.E.M error bars are shown. Incidence of HIF-2α localisation was statistically assessed between treatments using Fisher’s exact testing. Statistics on figures indicate relative to untreated control unless otherwise stated and * = *P* <0.05, ** = *P* <0.01, and *** = *P* <0.001.

## Results

### IL-1-induces reversible primary cilia elongation in a temporal, dose-dependent manner and indicative of altered ciliary trafficking

**Figure 1:**
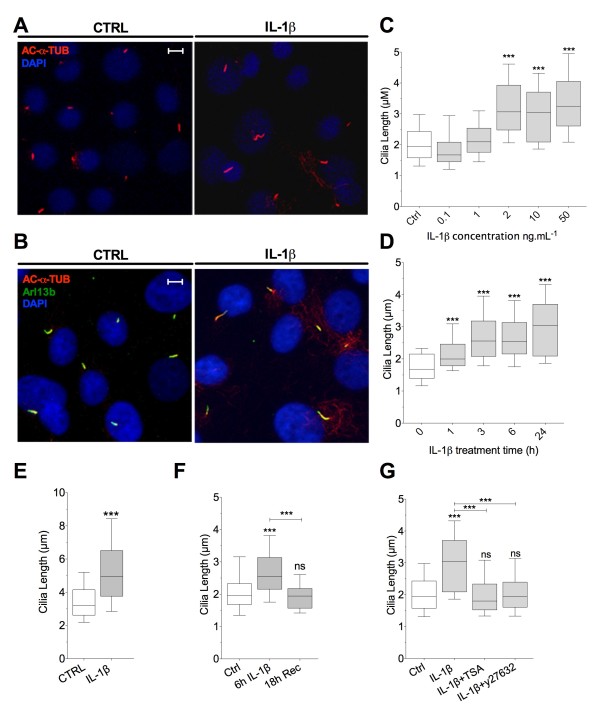
**Interleukin**-**1β induces dose dependent and reversible cilia elongation.** Confluent monolayers were treated with IL-1β before immunofluorescent staining of cilia. **(A)** Staining of cilia with anti-acetylated-α**-**tubulin and **(B)** concurrent anti-arl13b staining with 24 h IL-1β (10 ng.mL^-1^) treatment (scale =5 μm). **(C)** Bovine chondrocyte cilia length-IL-1 dose response at 24 h and **(D)** elongation time course at 10 ng.mL^-1^. **(E)** Ciliary elongation in human articular chondrocytes exposed to IL-1β (10 ng.mL^-1^, 24 h). **(F)** Reversibility of cilia elongation induced by IL-1β (10 ng.mL^-1^). **(G)** Inhibition of IL-1β induced-elongation with HDAC and ROCK inhibitors.

We first characterised the time-course and dose response effects of IL-1β on primary cilia length in bovine primary articular chondrocytes. The cilia structure was labelled with anti-acetylated alpha tubulin and visualised using confocal microscopy (Figure 1A/B, red). The membrane bound GTPase, ADP-ribosylation factor-like protein 13B (ARL-13b), was also found to be enriched in the chondrocyte cilium (Figure 1B, green) in agreement with other studies using other cell types [43]. ARL-13b was therefore used as an additional cilia marker. IL-1β treatment resulted in statistically significant increases in cilia length visualised using both cilia markers. However, in IL-1β-treated preparations ARL-13b expression appeared less homogenous, sometimes with large accumulations at the ciliary tip and regions with absence of staining in the axoneme, indicating alterations in ciliary trafficking. Therefore, cilia length data shown throughout this study are based on anti-acetylated alpha tubulin staining (Figure 1C onwards). In bovine articular chondrocytes statistically significant changes in cilia length occurred at 24 h, with concentrations of IL-1 β in excess of 1 ng.mL^-1^ (Figure 1C). The commonly used experimental concentration of IL-1β (10 ng.mL^-1^) induced slight elongation (19% increase in median) at 1 h (Figure 1D). Elongation was greater at 3 h (52% increase) but not maximised until 24 h treatment (81% increase). This increase at 24 h was statistically significantly different to increases seen at 1 h and 3 h, *P* = <0.0001 and 0.04, respectively. The elongation was reversible if the IL-1β (10 ng.mL^-1^) treatment media was gently removed after 6 h and replaced with control media left for a further 18 h (Figure 1F). In isolated human articular chondrocytes primary cilia length varied from 0.96 μm to 6.05 μm with a median value of 3.19 μm. IL-1β (10 ng.mL^-1^, 24 h) significantly increased human chondrocyte primary cilia length to a median value of 4.95 μm (*P* <0.0001, *n*= >100 cilia, (Figure 1E) representing a 55% increase. Cilia structure has been previously shown to be stabilised by inhibition of the activity of histone de-acetylase 6 (HDAC-6), present in the cilia axoneme [44,45]. We observe that cilia elongation induced by IL-1β (10 ng.mL^-1^, 24 h) was comprehensively blocked by concurrent treatment with the broad spectrum HDAC inhibitor Trichostatin A (TSA, 7 nM) or the Rho associated protein kinase (ROCK) inhibitor, Y27632 (10 μM) (Figure 1G). Neither TSA nor Y27632 had statistically significant effects on primary cilia length when used in the absence of IL-1β. These results indicate the IL-1 induced cilia elongation is dependent on both tubulin deacetylation and actin remodelling.

### IL-1 treatment increases HIF-2α expression

**Figure 2:**
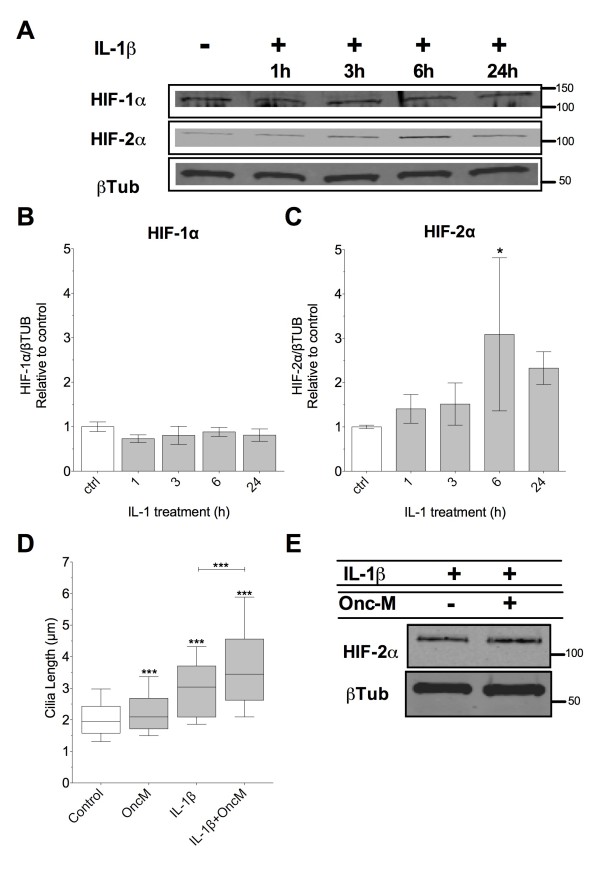
**Interleukin-1β increased HIF-2α expression.** Confluent preparations were treated with cytokines and total protein isolated. **(A)** Western blot analysis for HIFα expression during IL-1-treatment (10 ng.mL^-1^) time course. **(B/C)** Quantitative analysis of relative HIFα expression normalised to untreated control and β-tubulin. **(D)** Cilia length, as determined from immunofluorescent staining, with concurrent IL-1 and Oncostatin-M treatment, both at 10 ng.mL^-1^. **(E)** Western blot analysis of HIF2α expression with concurrent IL-1 and Oncostatin-M treatments.

Next we measured HIFα protein expression levels with IL-1β treatment using western blot. In primary bovine chondrocytes normoxic HIF-1α protein expression was low and appeared unaffected by IL-1β treatment within a 24 h period (Figure 2A/B). By contrast, HIF-2α expression gradually increased with 10 ng.mL^-1^ IL-1β treatment reaching statistical significance at 6 h before expression dropped down again at 24 h (*n* = 3, Figure 2A/C). The pathological effects of IL-1 in chondrocytes are often synergised by concurrent treatments with oncostatin-M, a member of the pro-inflammatory interleukin-6 (IL-6) family [46]. Additionally the catabolic effects of HIF-2α in cartilage have been attributed to IL-6 [47]. Therefore oncostatin-M was used to investigate the influence of IL-6 member inflammatory cytokines on cilia length and HIF expression. Oncostatin-M (10 ng.mL^-1^) had a small but statistically significant effect (8% increase in median) on cilia length in the absence of IL-1β. However, over a 24 h treatment IL-1β (10 ng.mL^-1^) in isolation produced a 57% increase in median cilia length but in the presence of oncostatin M this was increased to 77%; the difference being statistically significant (Figure 2D). This simultaneous treatment with IL-1 and oncostatin-M had no effect on HIF-2α expression (Figure 2E) indicating that elongation with oncostatin-M is independent of HIF-2α expression.

### HIF-2α is sequestered to the cilium by IL-1 treatment

**Figure 3:**
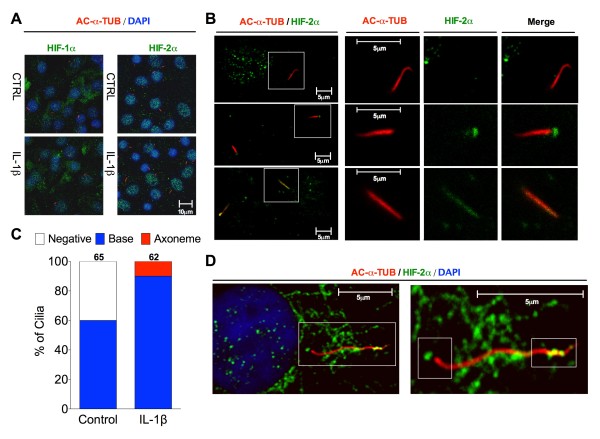
**HIF-2α accumulates in the cilium with IL-1β treatment. (A)** Whole cell HIFα expression (green), shown by immunofluorescent labelling, co-localised to nucleus (blue) in bovine chondrocytes. **(B)** Field images (large left-hand panels) and single cilium images (three small panels) showing varying degrees of HIF-2α expression in the cilium: negative cilia localisation (top), basal only localisation in samples treated with IL-1β (middle) and axonomal and basal staining with exposure to IL-1β (bottom). Single cilia images correspond to white boxes in field images. **(C)** Percentage of cilia exhibiting either basal only or axonomal and basal localisation of HIF-2α in untreated control cells or cells treated for 6 h with IL-1β (10 ng.mL^-1^), *n* shown above bars, fisher’s exact test statistics in text. **(D)** HIF-2α co-localised to primary cilium in human articular chondrocyte treated with IL-1β for 24 h.

HIFs are DNA-binding transcription factors that associate with specific nuclear co-factors to transactivate genes in order to respond to compromised oxygen tension. Consequently, both HIF-1α and HIF-2α are found predominantly in the nucleus as confirmed by co-localisation to nuclear DAPI staining (Figure 3A). No gross cytoplasmic re-localisation with IL-1β treatment was observed for either HIF-1α or HIF-2α (Figure 3A). However, in some cells HIF-2α was also found at the base of the primary cilium (Figure 3B). On closer inspection, this basal localisation was detectable in 59% of cells in untreated preparations. With IL-1β treatment, however, 100% of cilia robustly stained for HIF-2α, the difference being statistically significant (*P* = 0.0033, Fisher’s exact test). This was associated with an increased incidence of cells positive for HIF-2α expression at the primary cilia base (*P* = 0.0035, Fisher’s exact test, compared with untreated). Furthermore, in IL-1β-treated cells, 11% of cilia showed axonemal HIF-2α localisation, in addition to basal only expression. Cilia localisation data are summarised graphically in Figure 3C. *n* = 65 and 62 cilia for control and IL-1β groups, respectively. HIF-2α distribution was also assessed in human articular primary chondrocytes. While HIF-2α expression appeared higher in the cytoplasm of human cells than bovine, robust staining was observed at both the base and co-localised to acetylated alpha tubulin in the axoneme providing further evidence for HIF-2α ciliary trafficking (Figure 3D).

### Inhibition of HIF hydroxylases results in primary cilia elongation and is also associated with HIF-2α accumulation at the cilium

**Figure 4:**
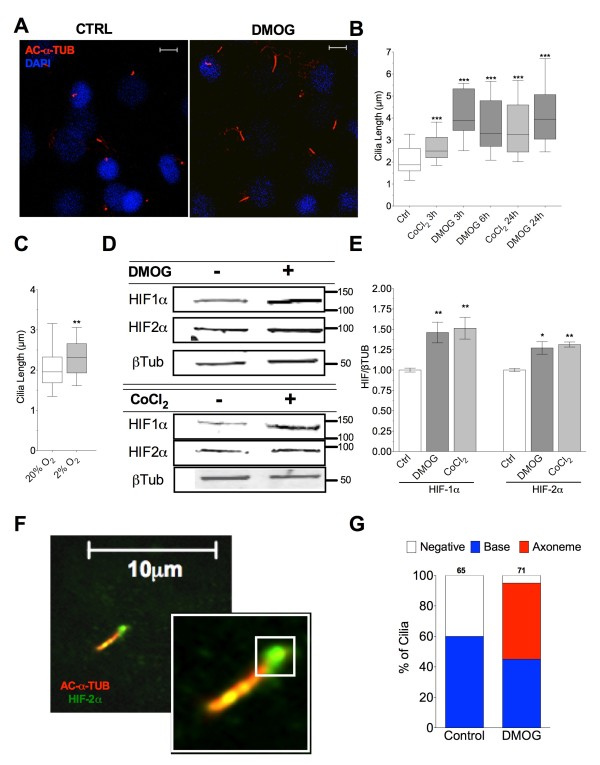
**Prolyl hydroxylase inhibition and hypoxia increases cilia length and is associated with accumulation of HIF-2α at the cilium. (A)** Immunofluorescent staining with anti-acetylated α tubulin with 24 h DMOG (10 μM) treatment (scale =5 μm). **(B)** Primary cilia length with prolyl hydroxylase inhibition. **(C)** Effect of 24 h hypoxia on primary cilia length. **(D/E)** Western blot analysis of HIFα expression after 24 h. **(F)** Immunofluorescent staining with anti-acetylated tubulin (red) and anti HIF-2α (green) in cells treated with DMOG. **(G)** Graphical summary of cilia localisation data for cells treated with DMOG, *n* above individual columns.

Dimethyloxallyl glycine (DMOG) is a competitive inhibitor of hif-α prolyl hydroxylase, thereby maintaining HIF-1α subunit expression in normoxia [48]. Cobalt chloride (CoCl_2_) is similarly used to maintain HIFα expression by inhibiting their hydroxylation and ultimate destruction by VHL and has been used previously as a hypoxia mimic and shown to influence cilia length [33]. Treatment with either DMOG (10 μM) or CoCl_2_ (100 μM) resulted in cilia elongation within 3 h, sustained to 24 h (Figure 4A/B). Most strikingly, cilia length doubled with 24 h DMOG treatment. An 18% increase in median cilia length was also observed in cultures placed at 2% oxygen for 24 h (Figure 4C). Both DMOG and CoCl_2_ modestly increased the total protein expression of HIF-1α and HIF-2α protein subunits, despite the presence of 20% oxygen, with 24 h treatment. This was assessed by western blotting (*n* = 3 for each treatment group) (Figure 4D/E). In DMOG treated preparations 95% of cilia exhibited ciliary HIF-2α staining (*P* = 0.019, Fisher’s exact test, compared with untreated control) with 50% of cilia showing HIF-2α in the axoneme (*P* = 0.006, Fisher’s exact test compared with IL-1β). A representative example of this staining is shown in Figure 4F. Cilia localisation data are again summarised graphically (Figure 4G), *n*= >65 and 71 cilia for control and DMOG groups, respectively.

### IL-1 induced primary cilia elongation is independent of increased HIF-2α expression

**Figure 5:**
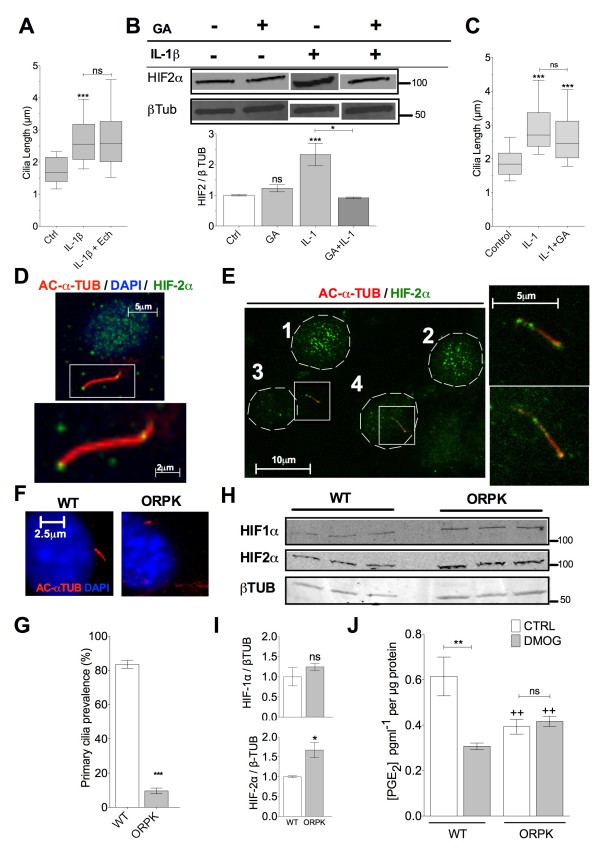
**Primary cilia elongation is independent of HIF-2α expression.** Loss of primary cilium results in elevated HIF-2α expression and altered response to DMOG. **(A)**  Antagonism of HIF binding to DNA had no effect on IL-1β-induced elongation. **(B)** Hsp90 inhibition with 7 nM GA inhibits IL-1-induced increases in HIF-2α expression but has no effect on IL-1β-induced cilia elongation **(C). (D)** HSP90 inhibition has no effect on ciliary HIF-2α which is still observed by immunofluorescent staining, in the presence of GA. **(E)** HIF-2α nuclear accumulation appears reduced in cells exhibiting primary cilia sequestering HIF-2α in response to IL-1. Hypomorphic mutation in the gene encoding for IFT88 in ORPK reduces the ability to build a primary cilium. **(F)** Immunofluorescent labelling of the primary cilium with anti-acetylated α tubulin in WT and ORPK cells. **(G)** Reduced primary cilia prevalence in ORPK. **(H)** Western blot analysis of HIF-1α and HIF-2α expression in WT and ORPK. **(I)** Quantification of western shows increase in HIF-2α expression in ORPK cells. **(J)** Altered response to DMOG in ORPK with respect to PGE_2_ production. ++ represents significant difference (*P* <0.01) from respective WT group.

The evidence so far indicates a temporal, biochemical and spatial relationship between HIF-2α and cilia structure such that the elongation seen with IL-1β is correlated with the recruitment of HIF-2α to the ciliary space. These observations are also made when cells are treated with DMOG, inhibiting HIF hydroxylation. We therefore tested whether HIFα activity and expression was required for IL-1-induced ciliary elongation. Addition of echinomycin (Ech) (1 μM), which blocks HIF binding to DNA [49], had no influence over IL-1β-induced elongation indicating the transcriptional activity of this protein was not required for this response (Figure 5A).

We next assessed the role of a candidate ciliary binding partner and regulator of HIF expression, the molecular chaperone, HSP90 [28,50]. This too was conducted in the context of IL-1-induced ciliary length change. Combined treatment of IL-1β (10 ng.mL^-1^) and HSP90 inhibitor 17-allylamino-17-demethoxygeldanamycin (GA, 7 nM) for 24 h reduced IL-1β-induced HIF-2α expression back to control levels (Figure 5B, *n* = 3 for protein expression). However, GA treatment did not affect cilia length compared with IL-1-treated preparations (Figure 5C). However, despite the reduced HIF-2α expression, ciliary localisation was still apparent in 75% of cells treated with both GA and IL-1 (Figure 5D). It was also noted that ciliary localisation was often, but not exclusively, correlated with an apparent reduction in nuclear localised HIF-2α compared with cells that did not express primary cilia (Figure 5E). Together these data indicated primary cilia elongation and the associated HIF-2α sequestration is independent of increases in HIF-2α expression.

### The loss of the primary cilium increases HIF-2α expression and alters PGE_2_ response to prolyl hydroxylase inhibition

Having observed qualitative reductions in nuclear HIF-2α associated with ciliary HIF-2α, we tested the hypothesis that HIF-2α is sequestered to the cilium in order to regulate HIF-2α expression and function. To do this we used a chondrocyte cell line (denoted Oak Ridge Polycystic Kidney (ORPK)) harbouring a hypomorphic insertional mutation in *TG737* encoding for polaris/IFT88 protein and resulting in reduced ciliation [15,41,42] (Figure 5F). Cilia prevalence was reduced from approximately 80% in WT cells to approximately 10% in mutant ORPK cells as a result of dysfunctional anterograde IFT88 (Figure 5G). Under normoxic conditions, where degradation pathways are most active, HIF-2α expression levels were elevated in ORPK cells compared with WT (*P* = 0.025, *n* = 3) (Figure 5H/I). No such statistically significant difference was observed in HIF-1α expression. The transcriptional targets of HIF-2α in chondrocytes have been the subject of some disagreement in the literature. Previously it has been reported that HIF-2 positively regulates SOX9 and downstream expression of aggrecan in chondrocytes [36]. We have previously reported ORPK cells to have increased aggrecan expression [15]. Another proposed target for HIF-2α in chondrocytes is prostaglandin endoperoxide synthase-2, the enzyme responsible for PGE_2_ production. In response to 24 h prolyl hydroxylase inhibition with DMOG (10 μM) PGE_2_ production is reduced in WT chondrocytes. This response is abolished in ORPK cells (J). These data suggest that the cilium and IFT exerts a negative influence over HIF-2α signalling at the level of its expression. This is associated with increases in gene targets of HIF-2α and alterations to the response to prolyl hydroxylase inhibition. To summarise both inflammatory stimuli and independent modulators of HIF-2α mediate an increase in cilia length which drives HIF-2α sequestration to the cilium. Furthermore, the data indicate the cilium negatively regulates HIF-2α expression and its downstream effects. Thus we propose that sequestration of HIF-2α to the cilium represents part of a post-translational feedback mechanism which may in turn regulate HIF-2α signalling during the response to inflammatory cytokines.

## Discussion

This study examined the link between primary cilia and HIFs in response to the inflammatory cytokine IL-1β. The study links previously described roles for the cilium in chondrocytes, including the regulation of matrix and IL-1 signalling [15,32], the effect of hypoxia on primary cilia length [33] and the biological roles of HIF-2α [29,36].

Within minutes of exposure, IL-1 is known to elicit early signalling events and subsequently activate NFκβ [51] inducing a plethora of cellular processes. In the present study IL-1β induced statistically significant primary cilia elongation at 1 h while more substantial elongation was observed from 3 h (Figure 1A). This implies elongation may be a gradual or adaptive response to an earlier activation of signalling pathways with maximal ciliary elongation at 24 h also dependant on protein translation and recruitment. We propose this elongation is reflective of increased net anterograde trafficking into the cilium, as seen in other ciliary elongation contexts [20] and indicated by changes in previously homogenous ARL-13b cilia staining in control samples. Given ARL-13b has established roles in IFT [52] it is likely that the contents of the cilium are also modulated by IL-1β treatment. The IL-1β ciliary response is reversible, highlighting the dynamic nature of any early elongation mechanisms. We show IL-1β-induced elongation is firmly dependent on Rho/ROCK activity. This is in agreement with other studies highlighting the underlying role for cytoplasmic actin in regulating cilia length [21]. Histone deacteylase (HDAC) activity, probably the tubulin deacetylase HDAC-6, is also required, perhaps in releasing ciliary tubulin from stabilising acetylation in order to alter structure either through its putative roles in arl GTPase activities [52] or through histone deacetylation and resultant alterations in gene expression.

In some agreement with the literature [29], we find that HIF-2α expression is increased by IL-1β treatment within a timeframe matching that of IL-1β-induced cilia elongation. However, this increase appears transient in nature such that it is most pronounced 6 h after IL-1β exposure with no statistically significant increase in expression at 24 h. We do not find such an effect on HIF-1α protein expression which was low and remained so in normoxic culture with IL-1β treatment.

We show for the first time that HIF-2α, a transcription factor found canonically in the nucleus, is also found located at the base of the primary cilium. This may imply HIF-2α trafficking through the basal body and or transition zone region is critical to the cilium’s influence [53]. Upon application of IL-1β and DMOG, this ciliary localisation of HIF-2α is increased such that the majority of cells are positive for HIF-2α at the cilia base and the transcription factor becomes accumulated in the cilia axonome. This suggests increased trafficking from the basal body into the ciliary compartment, or reduced ciliary exit, assuming localisation only becomes unequivocally apparent by microscopy when enhanced in magnitude. The oxygen sensitive prolyl hydroxylases are responsible for HIFα hydroxylation, targeting these subunits for subsequent destruction. Despite normoxic experimental conditions, the inhibition of these enzymes increases the expression of both HIFα subunits relative to untreated controls. Saliently both prolyl hydroxylase inhibitors used here, DMOG and CoCl_2_ elicit cilia elongation within 3 to 6 h of application despite exerting only subtle effects on HIF protein levels. Hypoxia itself also induces cilia elongation, albeit less dramatically, further linking HIFs to cilia length regulation and in concord with studies in kidney epithelia [33]. The physical recruitment of HIF-2α to the cilium indicated either a potential role for HIF-2α in modulating cilia structure or alternatively a role for the cilium in regulating the signalling or expression of HIF-2α. Our data indicate that despite the effects of prolyl hydroxylase inhibition and IL-1β upon cilia length, HIF-2α activity or expression does not cause ciliary elongation. When echinomycin (a DNA binding blocking agent for HIF-α) is added to IL-1β-treated preparations no influence on ciliary elongation was seen indicating that elongation does not depend on transcriptional HIFα activity. A binding partner for HIF-2α, in the form of HSP90, has previously been shown to be enriched in the cilium where it offers a structurally stabilising role to the cilium in the face of heat shock-mediated ciliary disassembly [44]. The binding relationship known to exist between HSP90 and HIFα leads to HIF stabilisation/induction [28] such that HSP90 deficiency or inhibition delays HIF accumulation. HSP90 inhibition with GA has been shown to potently inhibit HIF-2α expression [50] and in these studies reduced IL-1β-induced HIF2 expression to control levels thus abolishing IL-1β-induced increases in HIF-2α. Critically, however, cilia length changes with IL-1β were unaffected by GA treatment and ciliary localised HIF-2α was still observed indicating that trafficking to the cilium may be an ongoing event independent of expression levels. It does not appear that gross cellular HIF-2α expression regulates IL-1β-induced ciliary elongation but rather that IL-1-induced elongation is a result of increased anterograde trafficking, which enhances HIF-2α recruitment.

The trafficking of HIF-2α into the cilium may, therefore, represent an important regulation of HIF-2α. We propose that HIF-2α expression and transcriptional activity is regulated by the ciliary compartment. This proposal is supported by the finding that HIF-2α expression is elevated in ORPK cells where ciliogenesis is disrupted.

The biological roles of HIF-2α are still subject for debate, certainly in chondrocytes. Prolyl hydroxylase inhibition, raising HIF expression by either pharmacological means such as DMOG or hypoxic means, has been shown previously to be both pro- and anti-inflammatory but in chondrocytes hypoxia is proposed to be protective in response to inflammatory stimuli [30]. We find inhibition of PGE_2_ production in response to DMOG in WT cells is lost in ORPK cells, suggesting a role for the cilium in the response to prolyl hydroxylase regulation of HIF. Moreover, we have previously shown aggrecan, an established downstream target of HIF-2α, is upregulated in these cells [15] while others have shown prolyl hydroxylase inhibition to enhance matrix production [54]. In addition, IL-1β has been shown to negatively regulate matrix gene expression through downregulation of SOX9 [55].

**Figure 6:**
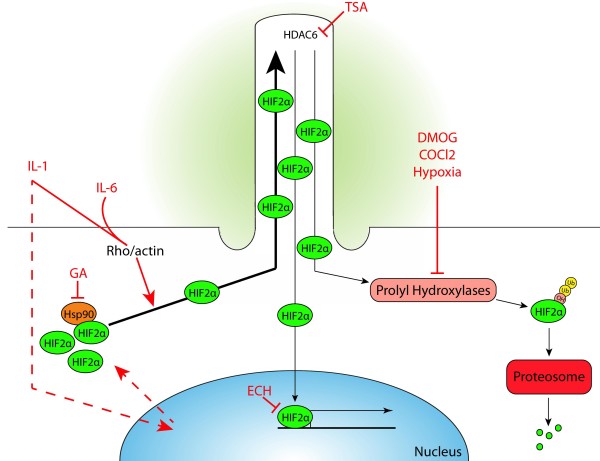
**Proposal for cilia role in the regulation of HIF2α expression/activity.** Interleukin-1 (IL-1) stimulates ciliary elongation through Rho-actin dependent modulation of anterograde intraflagellar trafficking (bold arrow, black). This elongation is enhanced in the presence of interleukin-6 (IL-6). Cilia elongation is also dependent on tubulin HDAC activity, most likely HDAC6, and can be blocked by trichostatin A (TSA). IL-1 triggers an initial increase in HIF-2α expression resulting in the sequestration of HIF-2α to the ciliary compartment. The inhibition of prolyl hydroxylases by DMOG, CoCl_2_ and hypoxia is also associated with cilia elongation and HIF-2α accumulation, possibly as part of a feedback mechanism such that accumulation of HIF-2a in the cilium drives further cilia elongation. However, HIF-2α is not required for cilia elongation as blockade of HIF transcriptional activity at the nucleus by echinomycin (ECH) has no effect on IL-1-induced elongation. Moreover, the Hsp90 inhibitor geldanamycin (GA) does not influence cilia elongation yet prevents the accumulation of HIF-2α downstream of IL-1. Removal of the cilium, by hypomorphic mutation of IFT88, results in an increase in HIF-2α protein levels suggesting the cilium exerts a negative influence over HIF2α. Consistent with this hypothesis, the IL-1-induced increase in HIF2α is diminished at later time points following ciliary sequestration. Thus we propose that the cilium functions as part of a negative feedback mechanism which influences the levels of HIF2α protein by modulating its proteasomal targeting and ultimate destruction.

Ciliary sequestration of transcription factors, to the detriment of nuclear entry and/or activity, is not without precedent as β-catenin is sequestered to the cilia compartment, downregulating canonical wnt signalling [5]. Additionally the functions of both Gli transcription factors [56] and STAT6 [57] are regulated by translocation to the cilium. Von Hippel Lindau protein (pVHL), the substrate recognition component of the E3 ubiquitin ligase complex that selectively polyubiquitinates prolyl-hydroxylated HIF-α subunits, has ciliary localisation [58]. This raises the possibility that the cilium is partially required as the locality for proteosomal targeting of HIF-2. This may form part of a feedback loop following inflammatory stimuli, whereby HIF-2α is sequestered to the cilium in order to target its degradation following vHL ubiquitination. This proposal is outlined in a summary schematic (Figure 6) which also seeks to summarise the findings of this study. Clearly further lengthy study is required to support this and starts with a requirement for understanding how HIF-2α ciliary localisation is regulated. There have been links made between the cilia compartment and proteosomal degradation before. This link involved the Bardet-Biedl syndrome (BBS) basal body proteins [59]. Intriguingly a study from 2008 indicates BBS4, involved in cargo targeting [60] is a candidate HIF-2α binding partner [61]. It may be through this interaction that HIF-2α is sequestered and future manipulation of this recruitment may be conducted in order to establish the broader repercussions of cilia HIF-2α recruitment.

## Conclusions

In summary, these studies strongly highlight the temporal, biochemical and importantly spatial relationship between HIF proteins, especially HIF-2α, and the cilium in the context of IL-1β signalling. For the first time we show HIF-2α is localised to the cilia base and recruited to the axoneme upon IL-1β exposure and inhibition of prolyl hydroxylases. Our data are consistent with the proposal that this recruitment to the primary cilium is involved in regulating the activity of HIF-2α. The study is the first to demonstrate primary cilia sequestration of HIF-2α and illuminate this potential new role for the cilium in HIF signalling during inflammation. Given the wide-ranging physiological and pathological roles for both HIFs and the primary cilium, the findings may have major implications in a variety of pathologies including arthritis and cancer, where HIFs and inflammation are implicated.

## Abbreviations

ARL13B: ADP-ribosylation factor-like protein 13b; BBS4: Bardet-Biedl syndrome protein 4; BSA: Bovine serum albumin; DAPI: 4',6-diamidino-2-phenylindole; DMOG: Dimethyloxallyl glycine; ECH: Echinomycin; GA: 17-(allylamino)17-demethoxygeldamycin; HDAC6: Histone deacteylase 6; HIF-2α: Hypoxia-inducible factor 2 alpha; HSP90: Heat shock protein 90; IFT: Intraflagellar transport; IGF: Insulin growth factor; IL-1β: Interleukin-1β; IL-6: Interleukin-6; NO: Nitric oxide; ONC-M: Oncostatin M; ORPK: Oak ridge polycystic kidney; PGE2: Prostaglandin E2; PBS: Phosphate buffered saline; PDGF: Platlet derived growth factor; ROCK: Rho associated kinase; TGF: Transforming growth factor; TSA: Trichostatin A; vHL: Von Hippel Lindau protein.

## Competing interests

The authors have no competing interests to declare.

## Authors’ contributions

AW designed and conducted all experiments. CT, PC and MK helped design experiments and were hugely influential throughout in adding intellectual content and critically assessing contents. Funding was acquired by PC and MK. All authors read and approved the final manuscript.
